# The use of anticoagulants in patients with non-valvular atrial fibrillation between 2005 and 2014: A drug utilization study using claims data in Japan

**DOI:** 10.1371/journal.pone.0203380

**Published:** 2018-09-05

**Authors:** Kiyoshi Kubota, Nobuhiro Ooba, Yukari Kamijima, Kuniyasu Sato, Daisuke Koide

**Affiliations:** 1 NPO Drug Safety Research Unit Japan, Tokyo, Japan; 2 Department of Clinical Pharmacy, Nihon University School of Pharmacy, Chiba, Japan; 3 Department of Biostatistics and Bioinformatics, Graduate School of Medicine, University of Tokyo, Tokyo, Japan; Providence VA Medical Center, UNITED STATES

## Abstract

**Background:**

Anticoagulant therapy is recommended in patients with atrial fibrillation (AF) but remains underused. The proper use of anticoagulants has been encouraged in guidelines frequently published over the past two decades.

**Materials and methods:**

In this study, we used insurance claims data collected from 2005 to 2014 to investigate the prevalence and incidence of non-valvular AF (NVAF) patients aged 20 to 74 years standardized to the Japanese population in 2012 and subdivided by stroke prevention drug type. We estimated the frequency of coagulation monitoring in patients with incident NVAF undergoing warfarin therapy in 2011 and later.

**Results:**

From 2005 to 2014, the standardized prevalence of NVAF increased from 117/100,000 to 278/100,000 and the proportion of anticoagulant users increased from 38.4% to 58.0%, while that of antiplatelet monotherapy decreased from 32.3% to 12.0%. The standardized incidence of NVAF was stable at ~40/100,000 patient-years. The proportion of those patients who started anticoagulant soon after the initial diagnosis increased from 19.9% to 49.1% from 2006 to 2013. Among patients who started warfarin, switchers to DOAC had more frequent coagulation monitoring than non-switchers.

**Conclusion:**

The use of anticoagulant therapy has gradually increased in patients with NVAF in Japan during the study period from 2005 to 2014.

## Introduction

Atrial fibrillation (AF) is a relatively common disease worldwide. According to estimates based on periodical health examinations in Japan, 0.56% of the population (approximately 700,000 persons) had AF in 2005 [1]. Despite anticoagulant therapy being recommended for the prevention of stroke and systemic embolism, it remains underused for patients with AF [2–15]. The main reasons for the underuse of anticoagulants include concerns about the risk of major bleeding associated with anticoagulant therapy and the need for frequent coagulation monitoring to maintain the target international normalized ratio (INR) inherent in warfarin therapy.

Anticoagulant therapy has been recommended for patients who have stroke risk factors while aspirin has been recommended for those with a low risk of stroke. For example, in one of the earliest guidelines on the prevention of a first stroke published in 1999 [16], warfarin was recommended for AF patients with high risk of stroke whereas aspirin was recommended for those aged under 65 years without any risk factors.

In the guidelines for the management of patients with AF published in 2001, warfarin was recommended for patients at high risk of stroke whereas aspirin in 325 mg daily doses was recommended for low-risk AF patients as well as patients with contraindications to anticoagulation [17]. Similar recommendations were made in drug treatment guidelines for AF published in 2001, 2008 and 2014 in Japan [18–20]. According to US guidelines published in 2014, aspirin was still described as one of the three possible options (aspirin, no antithrombotic therapy and oral anticoagulation) for antithrombotic therapy in patients with non-valvular AF (NVAF) who had a low risk of stroke (CHA_2_DS_2_-VASc score of 1) [21]. Only in the latest guidelines published in 2016 was antiplatelet monotherapy not recommended for stroke prevention in AF patients, regardless of stroke risk [22]. Therefore, the underuse of anticoagulants repeatedly observed in recent publications may imply that many patients who need anticoagulant therapy are using either antiplatelet only or no anticoagulant/antiplatelet at all. Nevertheless, guidelines frequently published during the past two decades concerning the use of anticoagulants in patients with NVAF suggest that a continuous effort has been made to improve treatment for stroke prevention in patients with AF.

The introduction of direct oral anticoagulant (DOAC) is another possible contributor to the better treatment of patients with AF. This is because the DOAC therapy does not involve frequent coagulation monitoring (one of possible reasons for the underuse of warfarin therapy) whereas the risk of bleeding associated with anticoagulation therapy using DOAC is, in general, not higher than that associated with warfarin [23–26] (except for concerns regarding the high risk of gastrointestinal bleeding in some patients with DOAC) [23,27].

In this study, we estimated trends in the use of antiplatelet and anticoagulants (warfarin and DOACs) in patients with NVAF between 2005 and 2014 in Japan.

## Materials and methods

We estimated the prevalence and incidence of patients with NVAF in a population aged between 20 and 74 years, inclusive, using insurance claims and enrolment from 2.8 million persons (about 2.3% of the total population in Japan). The collated data was obtained from the Japan Medical Data Center (JMDC), which maintains data from 40 corporate health insurance plans [28]. The insurance claims included information regarding the use of health care services, health conditions (diagnoses), the use of drugs and medical procedures. The inpatient claims had the same format as the outpatient claims and the information concerning drug use during hospitalization was obtained from the inpatient claims. The enrolment data included: year of birth, gender and dates when the individuals enrolled and unenrolled in the insurance plans. As the original data was provided to the JMDC under separate contracts with each of the individual insurers, the times at which data acquisition started and ended varied between the 40 insurance plans. The data acquisition started in January 2005 and ended in March 2014 for 4 insurance plans covering about 0.5 million persons, while the data acquisition commenced after January 2005 and/or ended earlier than March 2014 for the 36 insurance plans covering the remaining 2.3 million persons.

The claims data did not include the data of those aged 75 years or older as they were not covered by corporate health insurance but rather by the national “late-stage medical care system for the elderly” health insurance plan available to all citizens of Japan aged 75 years or older [29]. In addition, the proportion of the persons aged 65 to 74 years in the JMDC database was lower than that in the general population. For instance, in October 2012, only 4.2% of the persons aged 20 to 74 years were classed as elderly (i.e., 65 to 74 years of age) in the JMDC database despite persons in this age range comprising 17.4% of the population aged 20 to 74 years in Japan (according to the October 2012 census) [30]. Hence, to cope with the problems related to the skewed data due to the small proportion of elderly belonging to the corporate health insurance plans, the prevalence and incidence of patients with NVAF were standardized by age and gender to the general population in Japan. We elected to use the JMDC database because other data available in Japan did not include information from as early as 2005.

To identify patients with NVAF, we used local diagnosis codes. Local diagnosis codes are used in claims potentially with a "rule out" flag when the diagnosis is suspected but not yet confirmed. The diagnosis codes used in Japanese claims are officially maintained by the Medical Information System Development Center (MEDIS-DC) [31]. The MEDIS-DC also classifies the local diagnosis codes under one of the International Classification of Diseases 10th revision (ICD-10) codes. We found that 14 local codes in the database were located under the ICD-10 code "I48" (atrial fibrillation and flutter). They were local codes for "atrial fibrillation", "paroxysmal atrial fibrillation", "chronic atrial fibrillation", "atrial flutter", "non-valvular atrial fibrillation", "tachycardia atrial fibrillation", "persistent atrial fibrillation", "transient atrial fibrillation", "bradycardia atrial fibrillation", "non-valvular paroxysmal atrial fibrillation", "permanent atrial fibrillation", "tachyarrhythmia absoluta", "paroxysmal tachycardia atrial fibrillation" and "valvular atrial fibrillation".

A total of 11,593 patients had one of the 14 local diagnosis codes in their claims but only 7 patients (0.06%) had the code for "valvular atrial fibrillation" while 589 patients (5.1%) had the code for either "non-valvular atrial fibrillation" or "non-valvular paroxysmal atrial fibrillation". In the current study, we excluded 7 patients with the diagnosis code specified as "valvular AF" and the remaining patients were defined as "patients with NVAF". We then excluded patients whose NVAF diagnosis codes in the claims were all accompanied by the "rule out" flag. We further excluded patients who had a diagnosis code associated with potential indications of anticoagulant other than AF (i.e., pulmonary embolism, pulmonary hypertension, intracardiac thrombosis, arterial embolism/thrombosis, phlebitis/thrombophlebitis, portal vein thrombosis, venous embolism/thrombosis or presence of prosthetic heart valve) at or before the time when the first NVAF diagnosis code appeared.

**Fig 1:**
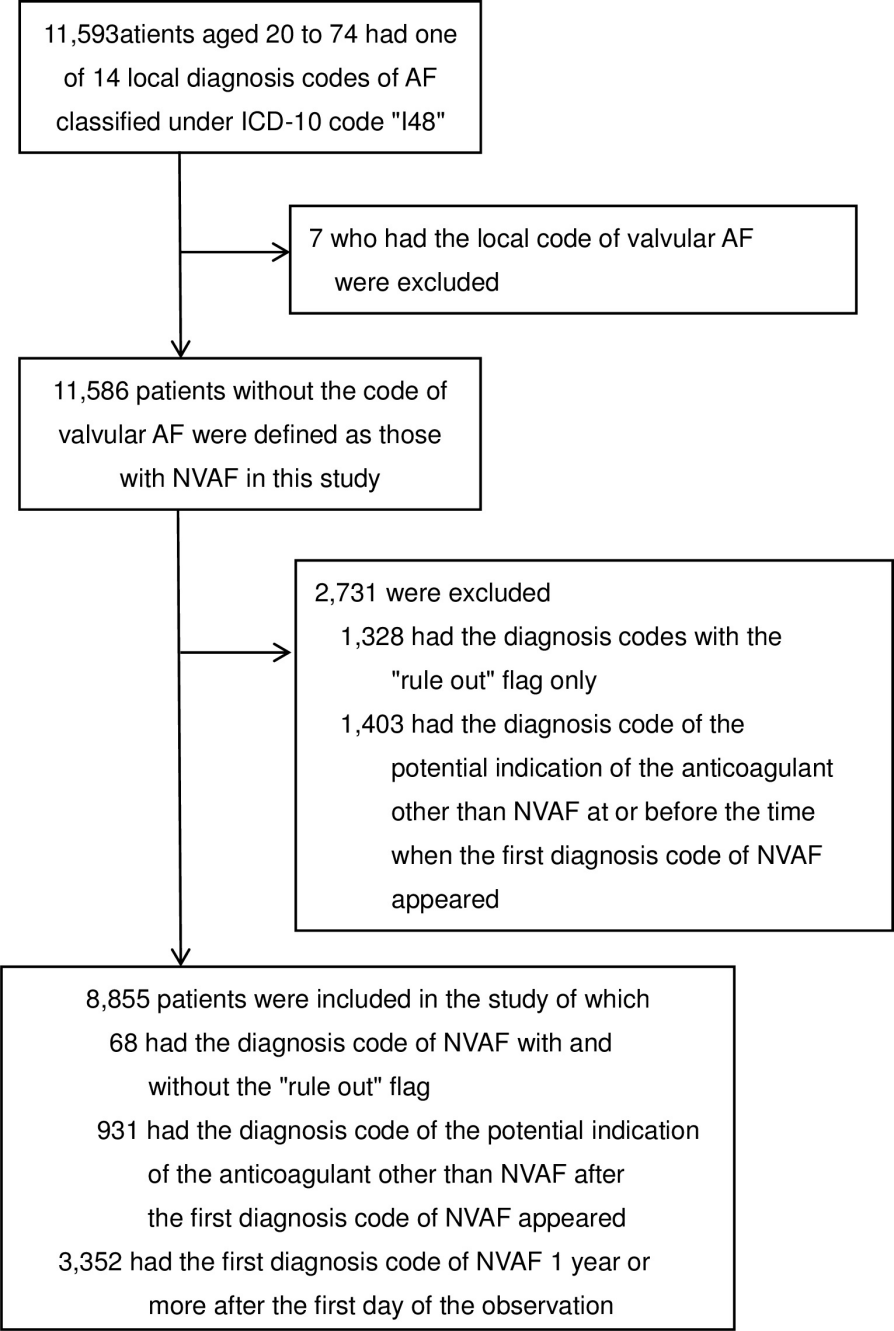
Study cohort.

**Fig 2:**
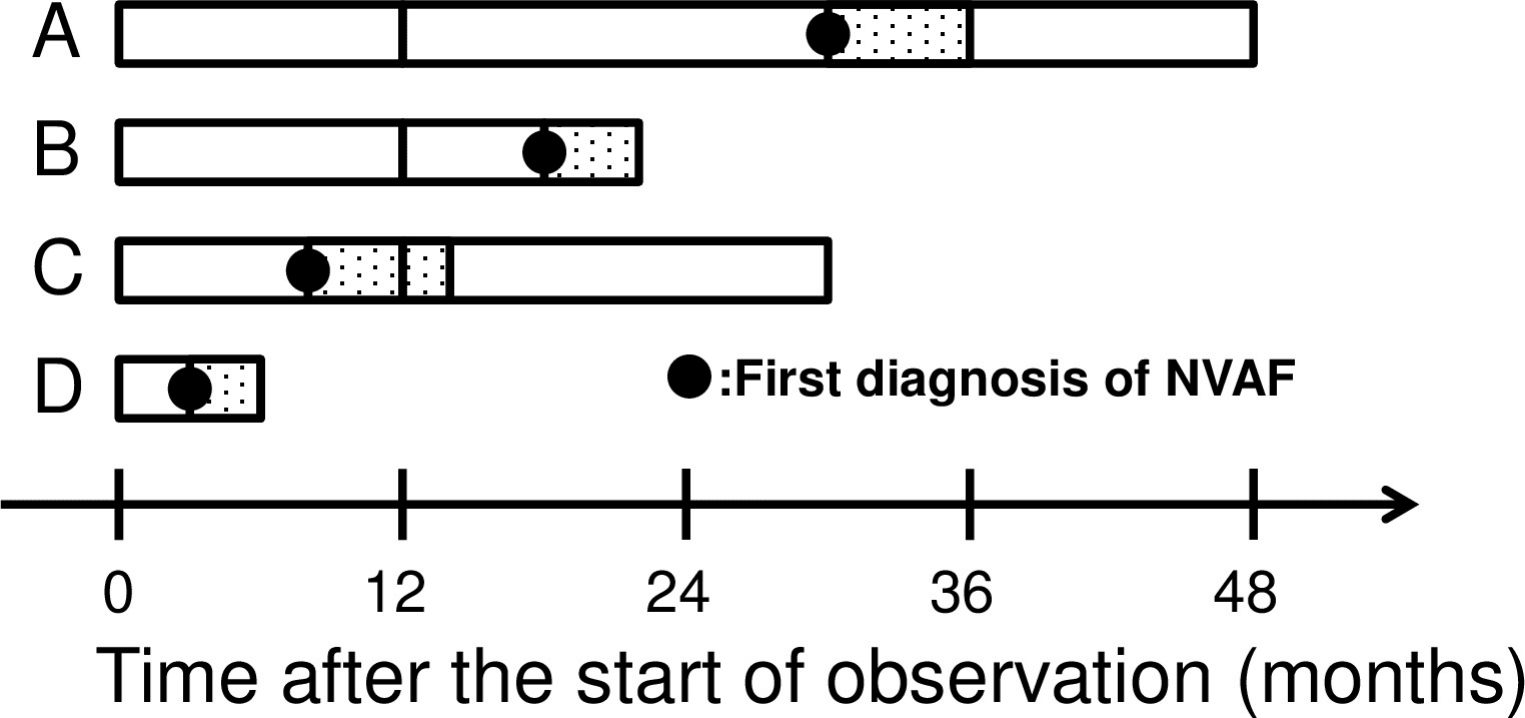
Classification of patients. Subjects A and B were classified as an incident NVAF patient as the first diagnosis code of NVAF appeared more than 1 year after the start of observation while subjects C and D were classified as a prevalent NVAF patient. In Drug Grouping II where patients were classified according the use of the drug during the 6 months after the first diagnosis of NVAF, subjects B and D were excluded because the observation period after the first diagnosis code of NVAF was less than 6 months.

Consequently, we obtained a total of 8,855 study subjects (Fig 1) who were further classified into two age groups: a "young" group of 7,451 patients aged 20–64 years old and an "old" group of 1,404 patients aged 65–74 years. The patients were also classified into 3,352 "incident" and 5,503 "prevalent" NVAF groups according to the time when the patient had the first diagnosis code of NVAF. In this classification, the patient was judged to have "incident" NVAF if the first diagnosis code of NVAF appeared 1 year or more after the start of observation period (defined as either the day that the person was enrolled, or the day that the data acquisition from the particular insurance commenced, whichever came later) while the patient was judged to have the "prevalent" NVAF otherwise (Fig 2).

We estimated the baseline characteristics of 3,352 incident NVAF patients using information during the 1 year preceding the first diagnosis code of NVAF. For 5,503 prevalent NVAF patients, the baseline characteristics were estimated using information during the first year of the observation period.

We calculated the prevalence of NVAF per quarter of each year. As NVAF is a potentially reversible condition, we counted the patient once per quarter when the patient had the condition code of NVAF (but without a "rule out" flag for that quarter), when the last day of the quarter preceded the last day of the observation period for the individual patient, and when the last day of the quarter preceded the first diagnosis code of a potential indication of anticoagulant other than NVAF.

The last day of the observation was defined as the day that either the person was unenrolled or when data acquisition from the particular insurance plan ended, whichever came earlier. To estimate the prevalence of NVAF, the patients in each quarter were classified according to the drug used in that quarter into the following four drug groups ("Drug Grouping I"): (1) “warfarin” when the patient had warfarin at least once but no DOAC; (2) “DOAC” when the patient had DOAC at least once irrespective of whether the patient also had warfarin; (3) “antiplatelet” when the patient had aspirin or clopidogrel at least once but had no anticoagulant; and (4) "no anticoagulant/antiplatelet" when the patient did not receive anticoagulant or antiplatelet.

In cases where dispensing of anticoagulant or antiplatelet commenced in a quarter when a patient also received a NVAF diagnosis code without a “rule out” flag, the patient was regarded to have the diagnosis code of NVAF up until the last day of the entire period of drug use (i.e., until the date of the dispensing plus days supply). Therefore, in such cases, the diagnosis of NVAF could be extended to subsequent quarters following the day of dispensing irrespective of whether a patient actually had the diagnosis code of NVAF in subsequent quarters. We estimated the prevalence, standardized by age (5-year band) and gender, by using the population in Japan aged between 20 and 74 years at the October 2012 census as the standard population (the "Standard Population Set").

We also calculated the proportion of the prevalence of the four drug groups for patients with NVAF in each quarter. To determine whether the advent of DOAC affected any trend in the use of anticoagulants, we conducted a segmented regression analysis [32] of the proportion of patients with anticoagulant (warfarin or DOAC) using Proc Autoreg in SAS 9.4 (SAS Institute, Cary, NC, USA) and the following model:

$$\text{Y}=\beta_{0}+\beta_{1}\;\text{time}+\beta_{2}\;\text{at the DOAC advent}+\beta_{3}\;\text{time after the DOAC advent}$$

where Y is the proportion of patients with a diagnosis code of NVAF who had anticoagulant (warfarin or DOAC), β_0_ is the intercept, β_1_ is the baseline time trend (where time was counted as the number of quarters after the observation started in the first quarter of 2005), β_2_ is the change in level at time of the DOAC advent in the first quarter of 2011 (when the first patient prescribed DOAC appeared), and β_3_ is the trend change after the DOAC advent.

In order to estimate the incidence of NVAF, the observation period was defined as the period from the first day of the observation until the last day of the observation. In estimating the incidence of NVAF, we excluded 32 patients who had the diagnosis code of NVAF with a "rule out" flag (as well as the code(s) without a "rule out" flag) from 3,352 incident NVAF patients as it was not easy to judge when these patients had the first NVAF.

We estimated the incidence rates of patients with incident NVAF of the following four mutually exclusive drug groups ("Drug Grouping II"): (1) "warfarin" when the patient had warfarin within 6 months after the first diagnosis of NVAF but before the patient had, if any, DOAC for the first time; (2) "DOAC" when the patient had DOAC within 6 months after the first diagnosis of NVAF but before the patient had, if any, warfarin for the first time; (3) "antiplatelet" if the patient had aspirin or clopidogrel but no warfarin or DOAC within 6 months after the first diagnosis of NVAF; and (4) "no anticoagulant/antiplatelet" when the patient did not receive any anticoagulant or antiplatelet within 6 months after the first diagnosis of NVAF.

We estimated the incidence of NVAF using 2,689 patients after excluding 631 incident NVAF patients. The observation period of those 631 ended within 6 months after the first diagnosis of NVAF (such as subject B in Fig 2) and therefore they were excluded so as not to misclassify them into a wrong drug group. Taking into account the exclusion of the 631 patients, when estimating the incidence rate of NVAF, the number of patient-years for each month was estimated by using the number of the insured in each month across the 40 insurance plans, observed for at least 1 year and then observed for at least further 6 months, then multiplied by one-twelfth.

We estimated the annual rate (per patient-year) of incident NVAF by dividing the total number of patients with incident NVAF during each year between 2006 and 2013 by the sum of the corresponding patient-years in the 12 months in any given year. The incidence rate was further standardized by age (5 year-band) and gender by reference to the Standard Population Set. We also estimated the proportion of incidence of each of the four drug groups classified according to "Drug Grouping II" across each year. When estimating the incidence of NVAF (but not in estimating the prevalence), if the first day of dispensing anticoagulant (but not antiplatelet) preceded the first day of the diagnosis code of NVAF, then the first day of the diagnosis of NVAF was "pushed forward" to the first day of dispensing anticoagulant to minimize the chance that a patient with prevalent NVAF was misclassified as a patient with incident NVAF.

To examine the duration of anticoagulant or antiplatelet therapy, we estimated the medication possession ratio (MPR) [33]. The incident NVAF patients were classified into four mutually exclusive groups according to "Drug Grouping II", and the MPR of anticoagulant and antiplatelet were estimated in the groups of “warfarin”, “DOAC” and “antiplatelet”, respectively. The MPR was estimated as the total of days supply (numerator) divided by the total of the period from the first day of dispending the drug until the last day of the observation period. The MPR was also estimated for prevalent NVAF patients to know whether the MPR in the prevalent NVAF patients was different from that in the incident NVAF patients.

Similar to incident NVAF patients, the prevalent NVAF patients were classified into four mutually exclusive groups of “warfarin”, “DOAC”, “antiplatelet” and “no anticoagulant/antiplatelet” according to "Drug Grouping II" regarding the anticoagulant or antiplatelet prescribed after (but within 6 months of) the first diagnosis code of NVAF. The prevalent NVAF patients were excluded from this analysis if the period between the first prescription of anticoagulant or antiplatelet and the last day the observation period was less than 6 months as for incident NVAF patients (such as subject D in Fig 2).

To establish the risk of stroke and bleeding, as well as the burden of comorbidities, the CHADS_2_ and CHA_2_DS_2_-Vasc scores for the risk of stroke [34,35], the HAS-BLED and ATRIA scores for the risk of bleeding [36,37] and the Charlson Comorbidity Index (CCI) score for the burden of comorbidities [38] were estimated for patients with incident NVAF using information gained from diagnoses and the use of drugs data during the year preceding the first diagnosis of NVAF. For prevalent NVAF patients, those scores were estimated using the information gained during the first year of the observation. The codes used to calculate those scores are shown in S1 File.

Lastly, to determine whether patients who had more frequent coagulation monitoring during warfarin tended to switch to DOAC compared with patients who had less frequent monitoring, we examined patients who had the incident NVAF in 2011 or later and then had warfarin. We divided those patients into patients who continued warfarin without switching to DOAC and those who subsequently switched to DOAC, and then compared the rate per year of measurements of prothrombin time during warfarin therapy.

For those who did not switch to DOAC, we estimated the rate during the period from the first date of dispensing of warfarin until either the last day of warfarin use (the date of the last dispensing of warfarin plus days supply) or the last day of the observation period, whichever came earlier. For those who switched to DOAC, we estimated the rate during the period of warfarin therapy defined as the period from the first date of dispensing of warfarin until 1 day before the first dispensing of DOAC.

This study was approved by the Ethics Committee of Nihon University School of Pharmacy (No. 14–011).

## Results

As shown in Fig 1, there were 8,855 study patients with NVAF; their mean age (SD) was 56.2 (12.3) years and 77.5% were males. They had a total of 64,663 quarters with a diagnosis code of NVAF without the "rule out" flag (7.3 quarters per patient). A total of 4,925 quarters were classified as "DOAC" and for 77.7%, 21.2% and 1.1% of these, the patients used dabigatran (marketed in March 2011), rivaroxaban (marketed in April 2012) and apixaban (marketed in February 2013), respectively.

Table 1 shows the baseline characteristics of 3,352 incident NVAF patients and 5,503 prevalent NVAF patients. The baseline characteristics in young (20 to 64 years of age) and old (65 to 74 years of age) patients are shown in S2 File. As shown in Table 1, the prevalent NVAF patients were older than the incident NVAF patients. The follow-up period of incident NVAF patients (whose observation period should be more than 1 year as for Subjects A and B in Fig 2) was longer than that in prevalent NVAF patients (whose observation period could be less than 1 year as for Subject D in Fig 2). The proportion of "missing" (i.e., the last day of observation of the individual patient before the last day of the data acquisition from the insurance due to various reasons including job change, retirement, reaching the age of 75 or death) in prevalent NVAF patients was higher than that in incident NVAF patients.

**Table 1:** Baseline characteristics of patient with incident and prevalent NVAF.

Variables	Incident NVAF ^a^	Prevalent NVAF ^b^
Population,N	3,352	5,503
Age, years (SD)	52.2 (11.4)	56.4 (10.4)
Male, (%)	77.4%	77.6%
Follow-up period, years, Mean (SD)	5.2 (2.5)	2.8 (2.0)
Median (IQR)	4.6 (3.2–7.2)	2.2 (1.3–4.2)
Missing, N(%) ^c^	680 (20.3%)	2,119 (38.5%)
Comorbidities (ICD-10 code), N (%) ^d^		
Malignant neoplasms (C0-C9)	209 (6.2%)	395 (7.2%)
Thyrotoxicosis (E05)	65 (1.9%)	409 (7.4%)
Diabetes mellitus (E10-E14)	621 (18.5%)	1,816 (33.0%)
Dyslipidemia (E78)	884 (26.4%)	2,414 (43.9%)
Hyperuricemia (E790)/ Gout (M10)	357 (10.7%)	1,180 (21.4%)
Depression (F32)	135 (4.0%)	280 (5.1%)
Insomnia (G47.0)	364 (10.9%)	968 (17.6%)
Hypertension (I10)	1,156 (34.5%)	3,531 (64.2%)
Ischemic heart disease (I20, I21)	446 (13.3%)	1,682 (30.6%)
Heart failure (I50)	338 (10.1%)	2,274 (41.3%)
Cerebral hemorrhage (I60, I61)	21 (0.6%)	42 (0.8%)
Cerebral infarction (I63)/ TIA (G45.9)	140 (4.2%)	712 (12.9%)
Acute upper respiratory infection (J06.9)	509 (15.2%)	909 (16.5%)
Allergic rhinitis (J30.4)	711 (21.2%)	1,211 (22.0%)
Asthma (J45)	331 (9.9%)	545 (9.9%)
Gastrointestinal bleeding (K22.6, K25.0, K25.4, K29.0)	31 (0.9%)	84 (1.5%)
Unspecified liver disease (K76.9)	204 (6.1%)	497 (9.0%)
Unspecified dermatitis (L30.9)	469 (14.0%)	805 (14.6%)
Chronic Kidney Disease (N18)	63 (1.9%)	148 (2.7%)
Co-medication, N (%) ^d^		
Antineoplastic drug	51 (1.5%)	87 (1.6%)
Antihyperthyroid drug	32 (1.0%)	219 (4.0%)
Oral antidiabetic drug	229 (6.8%)	667 (12.1%)
Insulin	90 (2.7%)	237 (4.3%)
Antihyperuricemic drug	259 (7.7%)	879 (16.0%)
Lipid lowering drug	511 (15.2%)	1,493 (27.1%)
Antidepressant	94 (2.8%)	205 (3.7%)
Anxiolytic drug	345 (10.3%)	862 (15.7%)
Antihypertensive drug	1,193 (35.6%)	3,938 (71.6%)
Loop diuretic	159 (4.7%)	792 (14.4%)
Antihistamine	904 (27.0%)	1,444 (26.2%)
Inhaled steroid	209 (6.2%)	311 (5.7%)
Anti-peptic ulcer drug	1,440 (43.0%)	2,901 (52.7%)
NSAID	1,346 (40.2%)	2,195 (39.9%)
Treatment for NVAF ^e^		
Warfarin	760 (22.7%)	2,242 (40.7%)
DOAC	496 (14.8%)	283 (5.1%)
Antiplatelet	725 (21.6%)	1,646 (29.9%)
Catheter ablation	177 (5.3%)	126 (2.3%)
Maze procedure	16 (0.5%)	6 (0.1%)
Drugs for rhythm control (iv) ^f^	371 (11.1%)	180 (3.3%)
Drugs for rhythm control (oral) ^g^	1,162 (34.7%)	1,595 (29.0%)
Scores for the risk of stroke, bleeding and burden comorbidities				
CHADS_2_ score (≥2)	991 (29.6%)	3,482 (63.3%)
CHA_2_DS_2_VASc score (≥2)	1,286 (38.4%)	4,008 (72.8%)
HAS-BLED score (≥3)	509 (15.2%)	1,515 (27.5%)
ATRIA score (≥3)	370 (11.0%)	900 (16.4%)
CCI score (≥3)	1,125 (33.6%)	2,751 (50.0%)

Abbreviations: NVAF, non-valvular atrial fibrillation; SD, standard deviation; IQR, interquartile range; ICD-10, 10th revision of the International Statistical Classification of Diseases; TIA, transient ischemic attack; NSAID, non-steroidal anti-inflammatory drug; DOAC, direct oral anticoagulant; iv, intravenous.

a. Patients who had the first diagnosis code of NVAF 1 year or more after the first day of observation period.

b. Patients who had the first diagnosis code of NVAF within 1 year after the first day of observation period.

c. Patients whose last day of observation was earlier than the last day when the data acquisition from the insurance ended.

d. Diagnoses or drugs recorded during the 1 year preceding the first diagnosis code of NVAF (for incident NVAF patients) or during the first year (for prevalent NVAF patients).

e. Treatment for NVAF recorded in the first 6 months after the first diagnosis code of NVAF where one patient may be counted twice or more for different treatments.

f. Solution of aprindine, amiodarone, cibenzoline, pilsicainide, flecainide or disopyramide for intravenous use.

g. Tablet or capsule of aprindine, amiodarone, cibenzoline, pilsicainide, flecainide, propafenone, bepridil or disopyramide for oral use.

The proportion of the patients with risk factors of AF (e.g., thyrotoxicosis and diabetes mellitus, hyperuricemia and heart failure) [22,39], diseases associated with high risk of stroke (e.g., hypertension, diabetes mellitus, heart failure and history of stroke) and that of the use of drugs to treat those diseases (e.g., antidiabetic drug, antihypertensive drug or loop diuretic) were higher in prevalent NVAF patients than those in incident NVAF patients.

As to the treatment for NVAF, the proportion of patients was higher for those receiving warfarin and antiplatelet but lower for DOAC in prevalent NVAF patients as compared to incident NVAF patients. The proportion of patients who underwent catheter ablation, maze procedure, or treatment with intravenous or oral use of drugs for rhythm control within 6 months after the first diagnosis of NVAF was higher in incident NVAF patients than in prevalent NVAF patients. All of CHA_2_DS_2_Vasc CHADS_2_, HAS-BLED, Atria and CCI scores were higher in prevalent NVAF patients than in incident NVAF patients.

**Fig 3:**
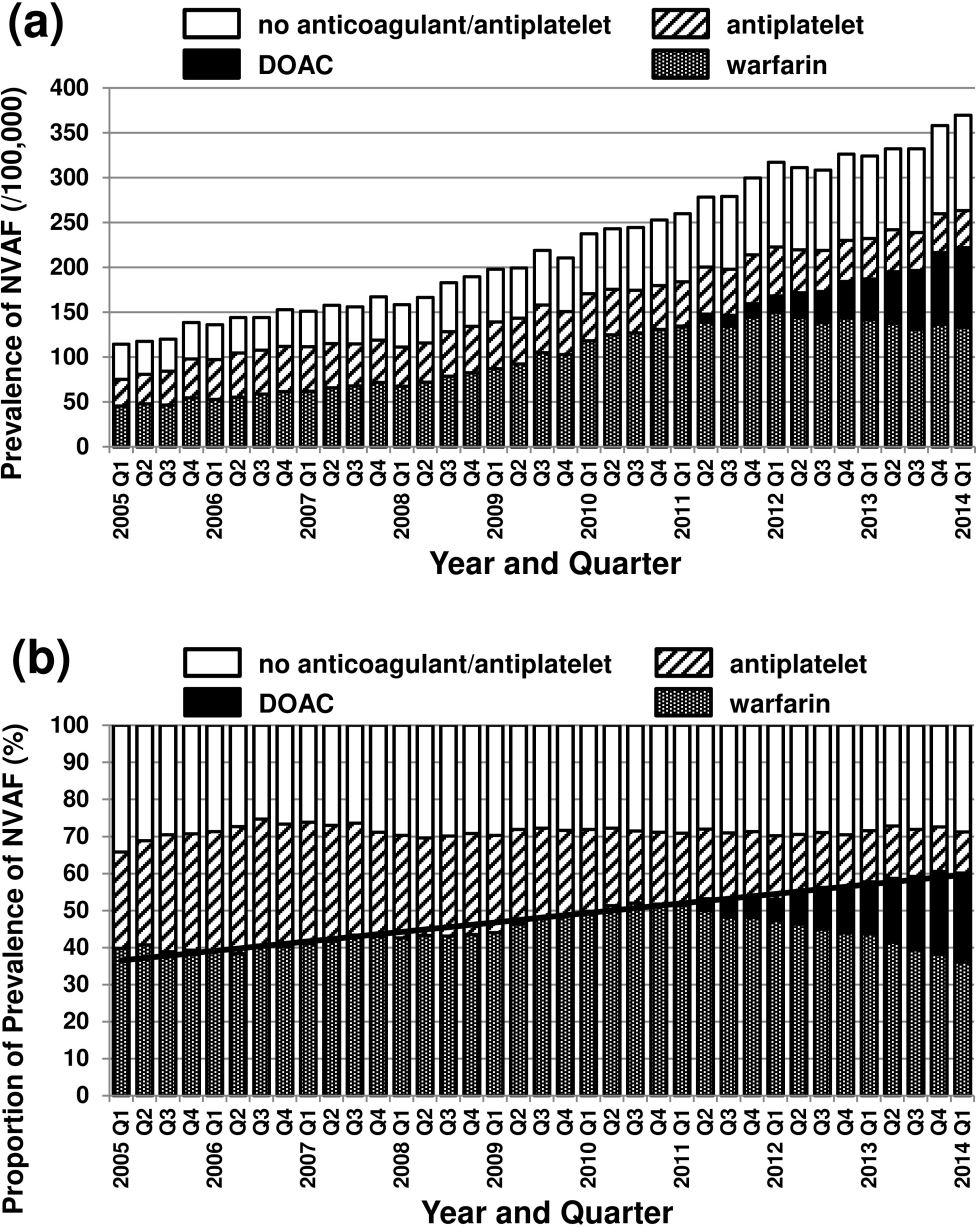
The prevalence of non-valvular atrial fibrillation (per/100,000 persons) age- and gender-standardized to Japanese population aged 20–74 years at the 2012 census (a) and the proportion of the prevalence subdivided by the use of the drug for stroke prevention (b) between the first quarter (Q1) of 2005 and the first quarter of 2014. DOAC: direct oral anticoagulant.

The standardized prevalence of patients with the diagnosis code of NVAF subdivided by anticoagulant/antiplatelet usage is shown in Fig 3A. The standardized prevalence was 117/100,000 in the first quarter (Q1) of 2005 but steadily increased to 278/100,000 in the first quarter of 2014. As shown in Fig 3B, during the period between the first quarter of 2005 and the first quarter of 2014, the proportion of patients with NVAF and anticoagulant (warfarin or DOAC) increased steadily from 38.4% to 58.0% while the proportion of those with antiplatelet decreased from 32.3% to 12.0%. The proportion of patients with NVAF with no anticoagulant/antiplatelet was ~30% throughout the period (Fig 3B).

During the period between the first quarter of 2005 and the first quarter of 2014, the increase in the standardized prevalence of patients with NVAF for the old patients (from 88/100,000 to 995/100,000) was more remarkable than that in the young patients (from 120/100,000 to 238/100,000). The increase of the proportion of the patients with anticoagulant in the old patients (42.7% to 66.7%) was also more remarkable than that in the young patients (from 39.3% to 54.3%) (S3 File).

In the segmented regression analysis conducted on the proportion of patients with anticoagulant (warfarin and DOAC), the Durbin-Watson test indicated an autocorrelation (p<0.0001). The results of the analysis using the autoregressive model with orders of 1 to 5 were similar to each other. The results for the second-order model are shown in Table 2. As both the level change (at the advent of DOAC) and the trend change (after the advent of DOAC) were not significant, a simple linear regression line is shown in Fig 3B. In both the young and old patients, the Durbin-Watson test also indicated an autocorrelation (p<0.0001) and the results of the analysis using the autoregressive model were essentially the same as those for all patients (S4 File).

**Table 2:** The results of the segmented regression analysis with the second-order autoregressive model for the proportion of the prevalence of patients with NVAF and anticoagulant (warfarin or DOAC) (N = 8,855).

Variables	Estimate	p value
Intercept (β_0_)	37.9	<0.0001
Baseline trend (β_1_)	0.547	<0.0001
Level change at DOAC advent (β_2_)	-0.128	0.91
Trend change after DOAC advent (β_3_)	0.238	0.27

Abbreviations: NVAF: non-valvular atrial fibrillation, DOAC: direct oral anticoagulant.

**Fig 4:**
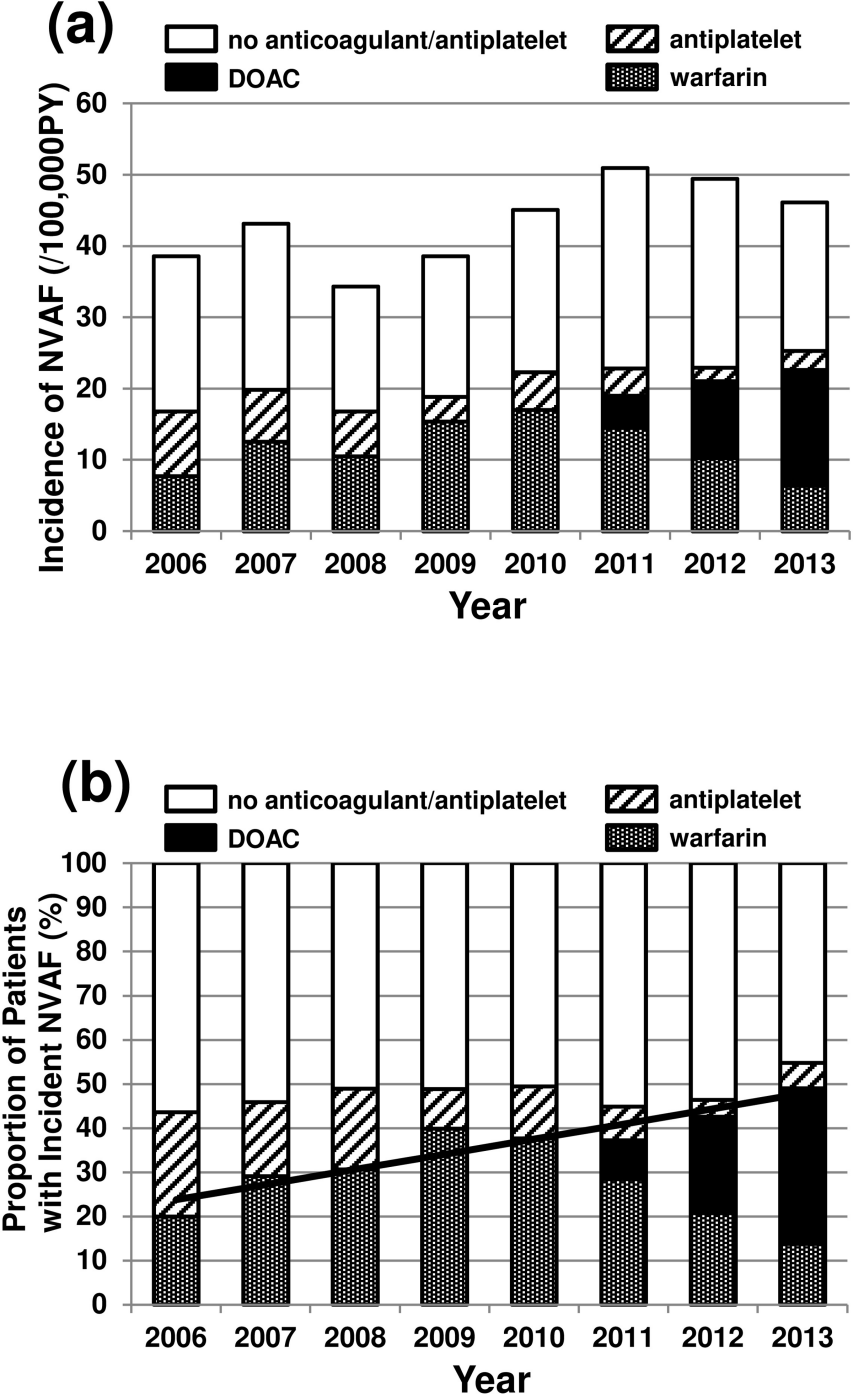
The incidence of non-valvular atrial fibrillation (per/100,000 person-years) age- and gender-standardized to Japanese population aged 20–74 years at the 2012 census (a) and the proportion of the incidence subdivided by the use of the drug for stroke prevention (b) between 2006 and the first quarter of 2013. DOAC: direct oral anticoagulant.

We found 2,689 patients with incident NVAF with no "rule out" flag. As shown in Fig 4A, the standardized incidence of NVAF was relatively constant at ~40/100,000 person-years during the period between 2006 and 2013. Fig 4B shows the proportion of patients with incident NVAF in the subgroups divided by the use of the drug starting within 6 months after the first diagnosis of NVAF for each year between 2006 and 2013. During the period between 2006 and 2013, the proportion of patients with anticoagulant (warfarin or DOAC) increased from 20.0% to 49.1%. In the young group, the incidence was also roughly constant, (~40/100,000 person-years), and during the period between 2006 and 2013, the proportion of the patients with anticoagulant increased from 22.7% to 44.3%. In the old group, the incidence increased from 40/100,000 to 81/100,000 person-years and the proportion of the patients with anticoagulant increased from 7.9% to 59.9% (S5 File).

Table 3 shows the medication possession ratio (MPR) and S6 File shows the information for the young (20 to 64 years of age) and old (65 to 74 years of age) patients. The MPR in the incident NVAF patients was lower than that in the prevalent NVAF patients. In both of the incident and prevalent NVAF patients, the MPR of antiplatelet was lower than that of anticoagulants. The MPR was similar between warfarin and DOAC for the incident NVAF patients but the MPR of warfarin was higher than that of DOAC in the prevalent NVAF patients.

**Table 3:** Medication possession ratio (MPR).

Class/Drug	N	Duration ^a^ (years)	MPR ^b^ (%)
Incident NVAF			
Antiplatelet	215	3.0	38.4
Warfarin	608	2.6	56.9
DOAC	317	1.2	58.9
Prevalent NVAF			
Antiplatelet	599	3.2	62.8
Warfarin	1,347	2.9	78.3
DOAC	138	1.4	70.4

Abbreviations: NVAF: non-valvular atrial fibrillation, DOAC, direct oral anticoagulant

a. Time duration from the first dispensing of the drug to the last day of observation (denominator).

b. Total days supply (numerator) divided by the total of the duration from the first dispensing to the last day of observation (denominator).

Table 4 shows the scores for the risk of stroke (CHADS_2_ and CHA_2_DS_2_VASc scores), the risk of bleeding (HAS-BLED and ATRIA scores) and the burden of the comorbidities (CCI score) of patients with incident NVAF. When patients were subdivided into three groups according to the calendar period when the patient first received a diagnosis code for NVAF, the risk in those receiving antiplatelet was roughly the same as those receiving warfarin but higher than those with no anticoagulant/antiplatelet across all of the calendar periods.

**Table 4:** The scores for the risk of stroke (CHADS_2_ score and CHA_2_DS_2_-VASc score) and bleeding (HAS-BLED score and ATRIA score) and the burden of comorbidities (CCI score) in patients with incident NVAF.

Period	January 2006 –June 2008			July 2008 –December 2010			January 2011 –September 2013			
Population, N	496,045			1,226,593			2,059,855			
Age, years (SD)	40.6(13.5)			40.2 (13.2)			40.7 (13.1)			
Male, %	58.3%			50.7%			52.0%			
New users	No AC/AP	AP	Warfarin	No AC/AP	AP	Warfarin	No AC/AP	AP	Warfarin	DOAC
Number	219	63	95	383	69	200	947	83	313	317
Age, years (SD)	49.5(10.7)	54.3(10.2)	53.7(9.5)	47.8(12.2)	53.6(11.5)	55.8(10.6)	50.0(11.3)	55.0(11.0)	54.9(10.1)	56.2(9.4)
Male, %	77.2%	81.0%	85.3%	73.6%	75.4%	82.5%	75.6%	79.5%	82.7%	82.3%
CHADS_2_ score (≥2), N %	42 19.2%	15 23.8%	23 24.2%	97 25.3%	25 36.2%	73 36.5%	226 23.9%	34 41.0%	114 36.4%	107 33.8%
CHA_2_DS_2_VASc score(≥2), N %	57 26.0%	20 31.7%	29 30.5%	124 32.4%	34 49.3%	91 45.5%	296 31.3%	43 51.8%	144 46.0%	134 42.3%
HAS-BLED score (≥3), N %	15 6.8%	12 19.0%	12 12.6%	45 11.7%	16 23.2%	42 21.0%	112 11.8%	15 18.1%	59 18.8%	46 14.5%
ATRIA score (≥3), N %	21 9.6%	3 4.8%	6 6.3%	33 8.6%	8 11.6%	17 8.5%	108 11.4%	10 12.0%	31 9.9%	30 9.5%
CCI score (≥3), N %	52 23.7%	17 27.0.%	18 18.9%	109 28.5%	26 37.7%	67 33.5%	320 33.8%	36 43.4%	105 33.5%	97 30.6%

Abbreviations: NVAF: non-valvular atrial fibrillation, SD: standard deviation, No AC/AP: no anticoagulant/antiplatelet; AP: antiplatelet; DOAC: direct oral anticoagulant; CCI: Charlson Comorbidity Index.

Patients with DOAC during the last calendar period (2011 or later) had a lower risk of stroke and bleeding as well as the lower burden of comorbidity as compared to those with warfarin or antiplatelet. Those findings (i.e., [i] that the risk of stroke/bleeding and the burden of the comorbidities were similar between three calendar periods for patients with warfarin and antiplatelet and [ii] the risk of stroke/bleeding and the burden of comorbidities were lower in those with DOAC than in those with warfarin or antiplatelet) were essentially the same in both the young and old groups although both the risk and the burden were, in general, higher amongst the old rather than in the young patients (S7 File).

Table 5 shows the rate of measurement of prothrombin time during warfarin therapy in 313 patients who had incident NVAF in 2011 or later and who were prescribed warfarin within 6 months. When they were subdivided into two groups, i.e., the 45 patients who switched to DOAC after they had warfarin and those 268 who did not, the rate of measurement of prothrombin time was higher for those who switched to DOAC than those who remained on warfarin therapy. When they were further divided into the young and old patients, the difference was larger in the old patients although the confidence interval was wide due to the small number of the old patients (S8 File).

**Table 5:** The rate of prothrombin time measurements during the warfarin therapy in patients with incident NVAF *.

	Patients N	Prothrombin time measurements N	Patient -years	Rate of measurement /patient-years
Warfarin followed by DOAC	45	430	30.0	14.4
Warfarin only	268	3,007	233.2	12.9
Rate difference (95% CI)				1.5 (0.0, 2.9)

*Data in 313 patients who had the first diagnosis code of NVAF in 2011 or later at least 1 year after the start of the observation period and then prescribed warfarin within 6 months.

## Discussion

The proportion of patients with NVAF receiving anticoagulant (warfarin or DOAC) was low (though it increased from 38.4% to 58.0% during the study period (Fig 3B)). Indeed, our study confirmed the underuse of the anticoagulant in patients with NVAF repeatedly observed and reported worldwide [2–15]. However, this proportion (38.4% to 58.0%) of patients had the diagnosis code of NVAF. If many patients with AF were not properly recognized as those with NVAF (i.e., the diagnosis code of NVAF was not given) in insurance claims, then the true proportion of patients (with or without the diagnosis code of NVAF in claims) with anticoagulant might be even lower.

In this study, we observed an increase in the prevalence of NVAF (Fig 3A). The increase was more remarkable in the old group than in the young group (Figure B in S3 File). It is not clear whether this increase was due to a true increase in the prevalence of NVAF or due to an increase in the proportion of patients with NVAF who were regarded as requiring medical care (and therefore assigned a diagnosis code of NVAF in insurance claims). However, the latter explanation is more likely because the incidence of NVAF (the rate of the appearance of patients with the first diagnosis code of NVAF 1 year or more after the start of the observation) was roughly constant during the study period, as shown in Fig 4A. If the true prevalence of patients with NVAF was stable during the study period between 2005 and 2014, then the increase of the proportion of patients with NVAF with anticoagulant therapy would be much faster than that suggested in Fig 3B (i.e., 1.5 times increase from 38.4 to 58.0%) and better represented in the increase of the prevalence of patients with anticoagulant as shown in Fig 3A (i.e., a greater than four times increase during the study period).

In the old group, we observed a two-fold increase in the incidence of NVAF (Fig B in S5 File), which may have also contributed to the ~10-fold increase in the prevalence during the period between 2005 and 2014 (Figure B in S3 File). Again, it is not clear whether the two-fold increase of incidence in the old group might indicate a true increase in the incidence of NVAF, or be merely due to an increase in the number of old patients with NVAF who were newly recognized as requiring medical care. We believe, however, that the latter could provide a more reasonable explanation than the former because the standardized prevalence of NVAF in the first quarter of 2005 in the old group (88/100,000) was lower than that for the young group (120/100,000), while the prevalence of NVAF should be higher in the old than that in the young.

It is universally observed that the prevalence of NVAF in an old group is higher than that in a young group although the difference in the prevalence between the old and young is not so remarkable in Japan compared to Western countries. For example in a study published in 2009 in Japan, the prevalence of AF in age groups 40–59, 60–69 and 70–79 years was 0.8%, 1.9% and 3.4% in men and 0.1%, 0.4% and 1.1% in women, respectively [40].

In the segmented regression analysis, we found that the advent of DOAC did not have major effects on the trend in the use of anticoagulant (Table 2). However, we also found that the risk of stroke and bleeding as well as the burden of comorbidities in those with DOAC were lower than those with warfarin. In addition, the rate of measurement of prothrombin time during warfarin therapy was higher in patients with incident NVAF in 2011 or later in those who subsequently switched to DOAC compared to those patients who continued warfarin treatment (Table 3). Therefore, the introduction of DOAC may have encouraged the use of anticoagulant therapy in low-risk patients and in those requiring frequent coagulation monitoring.

The current study has several limitations. First, our data did not include patients aged 75 years or older and therefore we could not assess drug use trends in that age group during the study period. Even among those aged less than 75 years, the data were skewed due to the lower proportion of the old group (65 to 74 years of age) compared with the general population. To overcome this problem, the prevalence and incidence of NVAF were standardized by age and gender to estimate the figures in the general population. In addition, the data used in this study came from corporate insurance plans where workers in relatively large firms (and their dependents) were insured and we cannot postulate whether our finding could be generalized to cover those insured by other health insurance plans. However, the fee for medical care is the same nationwide irrespective of insurance plan [41], and it is unlikely that medical care varies substantially between them. Furthermore, the accuracy of the diagnosis code of NVAF has not been previously examined in validation studies in Japan.

Our study also has some strengths. The data was collected during a relatively long period (~9 years). Furthermore, information concerning the use of drugs during hospitalization is often difficult to obtain, as observed in some database studies conducted in North America or Europe [42], however we managed to gain access to data on the use of drugs by inpatients as well as outpatients. Therefore, our study provided accurate estimates concerning drug use in both outpatients and inpatients during the study period.

## Conclusions

We observed a constant increase in the number of patients with NVAF treated by anticoagulant during the period between 2005 and 2014 in Japan. The increase of the prevalence was probably mainly due to the increase of patients with NVAF recognized as requiring medical care rather than the true increase of patients in the general population. The increase in the proportion of patients with NVAF treated by warfarin took place before the introduction of DOAC. However, the introduction of DOAC contributed to an increased use of anticoagulant in low-risk patients as well as those with frequent coagulation monitoring during warfarin therapy. These findings provide a foundation for future studies on both the beneficial and harmful effects of warfarin and DOAC in Japan.

## Supporting information

S1 File Codes used to estimate scores to measure the risk of stroke and bleeding, and the burden of comorbidities using Japanese claims. (DOCX)

S2 File Baseline characteristics of patient with incident and prevalent NVAF in age groups. Table A. Young (20–64 years old) patients (N = 7,451). Table B. Old (65–74 years old) patients (N = 1,883).
(DOCX)

S3 File The prevalence of non-valvular atrial fibrillation (NVAF) and the proportion of the prevalence in age groups. Figure A. Young (20–64 years old) patients (N = 7,451). Figure B. Old (65–74 years old) patients (N = 1,883).
(DOCX)

S4 File The results of the segmented regression analysis with the second-order autoregressive model for the proportion of the prevalence of patients with non-valvular atrial fibrillation (NVAF) and anticoagulant (warfarin or direct oral anticoagulant (DOAC)) in age groups. Table A. Young (20–64 years old) patients (N = 7,451). Table B. Old (65–74 years old) patients (N = 1,883).
(DOCX)

S5 File The incidence of non-valvular atrial fibrillation (NVAF) and the proportion of the prevalence in age groups. Figure A. Young (20–64 years old) patients (N = 2,403). Figure B. Old (65–74 years old) patients (N = 286). Figure A_a and Figure B_b show the incidence (/100,000 person-years) standardized to Japanese population as of the 2012 census. Figure A_b and Figure B_b show the proportion of the incidence subdivided by the drug started within 6 months after the first diagnosis of NVAF (%).
(DOCX)

S6 File The medication possession ratio (MPR) of anticoagulant and antiplatelet in incident and prevalent NVAF patients in age groups. Table A. Young (20–64 years old) patients (N = 7,451). Table B. Old (65–74 years old) patients (N = 1,883).
(DOCX)

S7 File The scores for the risk of stroke (CHADS_2_ score and CHA_2_DS_2_-VASc score) and bleeding (HAS-BLED score and ATRIA score) and the burden of comorbidities (CCI score) in incident NVAF patients in age groups. Table A. Young (20–64 years old) patients (N = 2,403). Table B. Old (65–74 years old) patients (N = 286).
(DOCX)

S8 File The rate of prothrombin time measurements during the warfarin therapy in age groups. Table A. Young (20–64 years old) patients (N = 263). Table B. Old (65–74 years old) patients (N = 50).
(DOCX)
