# Supplementary material for: The use of anticoagulants in patients with non-valvular atrial fibrillation between 2005 and 2014: A drug utilization study using claims data in Japan
Source: PLoS One. 2018 Sep 5;13(9):e0203380. doi: 10.1371/journal.pone.0203380 (PMC6124773; doi:10.1371/journal.pone.0203380)
Supplement: S1 File — (DOCX) [file pone.0203380.s001.docx]

S1 File.

CHADS_2_ score

CHF/LV dysfunction (1): All local diagnosis codes under ICD-10 code “I50” (Heart failure).

Hypertension (1): All local diagnosis codes under ICD-10 code “I10” (Essential hypertension), “I11” (Hypertensive heart disease), “I12” (Hypertensive renal disease), “I13” (Hypertensive heart and renal disease) and “I15” (Secondary hypertension).

Age 75 or over (1): estimated from age at the first diagnosis of NVAF.

Diabetes Mellitus (1): All local diagnosis codes under ICD-10 code “E10” (Type 1 diabetes mellitus), “E11” (Type 2 diabetes mellitus), “E13” (Other specified diabetes mellitus) and “E14” (Unspecified diabetes mellitus).

Stroke/TIA (2): All local diagnosis codes under ICD-10 code “I60” (Subarachnoid haemorrhage), “I61” (Intracerebral haemorrhage), “I62” (Other nontraumatic intracranial haemorrhage), “I63” (Cerebral infarction), “I64” (Stroke, not specified as haemorrhage or infarction), “I65” (Occlusion and stenosis of precerebral arteries, not resulting in cerebral infarction), “I66” (Occlusion and stenosis of cerebral arteries, not resulting in cerebral infarction), “I67” (Other cerebrovascular diseases), “I68” (Cerebrovascular disorders in diseases classified elsewhere), “I69” (Sequelae of cerebrovascular disease), “G45” (Transient cerebral ischaemic attacks and related syndromes) and “G46” (Vascular syndromes of brain in cerebrovascular diseases).

CHA_2_DS_2_-Vasc score

CHF/LV dysfunction (1): All local diagnosis codes under ICD-10 code “I50” (Heart failure).

Hypertension (1): All local diagnosis codes under ICD-10 code “I10” (Essential hypertension), “I11” (Hypertensive heart disease), “I12” (Hypertensive renal disease), “I13” (Hypertensive heart and renal disease) and “I15” (Secondary hypertension).

Age 65-74 (1) and Age 75 or over (2): estimated from age at the first diagnosis of NVAF.

Diabetes Mellitus (1): All local diagnosis codes under ICD-10 code “E10” (Type 1 diabetes mellitus), “E11” (Type 2 diabetes mellitus), “E13” (Other specified diabetes mellitus) and “E14” (Unspecified diabetes mellitus).

Stroke/TIA (2): All local diagnosis codes under ICD-10 code “I60” (Subarachnoid haemorrhage), “I61” (Intracerebral haemorrhage), “I62” (Other nontraumatic intracranial haemorrhage), “I63” (Cerebral infarction), “I64” (Stroke, not specified as haemorrhage or infarction), “I65” (Occlusion and stenosis of precerebral arteries, not resulting in cerebral infarction), “I66” (Occlusion and stenosis of cerebral arteries, not resulting in cerebral infarction), “I67” (Other cerebrovascular diseases), “I68” (Cerebrovascular disorders in diseases classified elsewhere), “I69” (Sequelae of cerebrovascular disease), “G45” (Transient cerebral ischaemic attacks and related syndromes) and “G46” (Vascular syndromes of brain in cerebrovascular diseases).

Vascular disease (1): All local diagnosis codes under ICD-10 code “I21” (Acute myocardial infarction), “I22” (Subsequent myocardial infarction), “I25” (Chronic ischaemic heart disease) and “I70” (Atherosclerosis).

Female (1): the information on gender was used.

HAS-BLED score

Hypertension (1): All local diagnosis codes under ICD-10 code “I10” (Essential hypertension), “I11” (Hypertensive heart disease), “I12” (Hypertensive renal disease), “I13” (Hypertensive heart and renal disease) and “I15” (Secondary hypertension).

Abnormal kidney function (1): All local diagnosis codes under ICD-10 code “N01” (Rapidly progressive nephritic syndrome), “N03” (Chronic nephritic syndrome), “N05” (Unspecified nephritic syndrome), “N07” (Hereditary nephropathy, not elsewhere classified), “N18” (Chronic kidney disease), “N19” (Unspecified kidney failure), “N25” (Disorders resulting from impaired renal tubular function) and “N28” (Other disorders of kidney and ureter, not elsewhere classified).

Note: We did not use a local code of “renal function test abnormal” (20066221) located under “R94.4” (Abnormal results of kidney function studies) as the original description (Chest 2010; 138: 1093-1100) for “abnormal kidney function” (“presence of chronic dialysis, renal transplantation, or serum creatinine ≥ 200  mol/L”) was not considered to include mild abnormal renal function test.

Abnormal liver function (1): All local diagnosis codes under ICD-10 code “K70” (Alcoholic liver disease), “K71” (Toxic liver disease), “K72” (Hepatic failure, not elsewhere classified), “K73” (Chronic hepatitis, not elsewhere classified), “K74” (Fibrosis and cirrhosis of liver) “K75” (Other inflammatory liver diseases) and “K76” (Other diseases of liver).

Note: We did not use a local code of “liver function test abnormal” (20057054) located under “R94.5” (Abnormal results of liver function studies) as the original description (Chest 2010; 138: 1093-1100) for “abnormal liver function” (“chronic hepatic disease (eg, cirrhosis) or biochemical evidence of significant hepatic derangement (eg, bilirubin > 2 X upper limit of normal, in association with aspartate aminotransferase/alanine aminotransferase/ alkaline phosphatase > 3 X upper limit normal, and so forth) was judged not to include mild abnormal liver function test.

Stroke (1): All local diagnosis codes under ICD-10 code “I60” (Subarachnoid haemorrhage), “I61” (Intracerebral haemorrhage), “I62” (Other nontraumatic intracranial haemorrhage), “I63” (Cerebral infarction), “I64” (Stroke, not specified as haemorrhage or infarction), “I65” (Occlusion and stenosis of precerebral arteries, not resulting in cerebral infarction), “I66” (Occlusion and stenosis of cerebral arteries, not resulting in cerebral infarction), “I67” (Other cerebrovascular diseases), “I68” (Cerebrovascular disorders in diseases classified elsewhere) and “I69” (Sequelae of cerebrovascular disease).

Bleeding (1): All local diagnosis codes under ICD-10 code “D62” (Acute posthaemorrhagic anaemia), “I61” (Intracerebral haemorrhage), “I62” (Other nontraumatic intracranial haemorrhage), “I85.0” (Oesophageal varices with bleeding), “J94.2” (Haemothorax), “K22.6” (Gastro-oesophageal laceration-haemorrhage syndrome), “K25.0”, “K25.2”, “K25.4”, “K25.6” (Gastric ulcer with bleeding), “K26.0”, “K26.2”, “K26.4”, “K26.6” (Duodenal ulcer with bleeding), “K27.0”, “K27.2”, “K27.4”, “K27.6” (Peptic ulcer, site unspecified with bleeding), “K28.0”, “K28.2”, “K28.4”, “K28.6” (Gastrojejunal ulcer with bleeding), “K29.0” (Acute haemorrhagic gastritis) , “K62.5” (Haemorrhage of anus and rectum), “K66.1” (Haemoperitoneum), “K92.0” (Haematemesis), “K92.1” (Melaena), “K92.2” (Gastrointestinal haemorrhage, unspecified),　“M25.0” (Haemarthrosis), “N42.1” (Congestion and haemorrhage of prostate), “N92.0” (Excessive and frequent menstruation with regular cycle), “N92.1” (Excessive and frequent menstruation with irregular cycle), “R04” (Haemorrhage from respiratory passages), “R31” (Unspecified haematuria), “R58” (Haemorrhage, not elsewhere classified), “S06.4” (Epidural haemorrhage), “S06.5” (Traumatic subdural haemorrhage), “S06.6” (Traumatic subarachnoid haemorrhage) and “S06.8” (Other intracranial injuries).

Liable INR (1): no patient was relevant as the information before the patient started the anticoagulant therapy was used to calculate the score.

Elderly (> 65 of age) (1): estimated from age at the first diagnosis of NVAF.

Anticonagulant/NSAID (1): Aspirin, clopidogrel or all drugs under ATC code “M01A” (antiinflammatory and antirheumatic products, non-steroids)

Alcoholism (1): All local diagnosis codes under ICD-10 code “F10” (Mental and behavioural disorders due to use of alcohol), “G31.2” (Degeneration of nervous system due to alcohol), “G62.1” (Alcoholic polyneuropathy), “G70.1” (Toxic myoneural disorders), “G72.1” (Alcoholic myopathy), “I42.6” (Alcoholic cardiomyopathy), “K70” (Alcoholic liver disease) and “K86.0” (Alcohol-induced chronic pancreatitis).

ATRIA score

Anemia (3): All local diagnosis codes under ICD-10 code “D50” (Iron deficiency anaemia) and “D64.9” (Other anaemias).

Severe renal disease (3): All local diagnosis codes under ICD-10 code “N01” (Rapidly progressive nephritic syndrome), “N03” (Chronic nephritic syndrome), “N05” (Unspecified nephritic syndrome), “N07” (Hereditary nephropathy, not elsewhere classified), “N18” (Chronic kidney disease), “N19” (Unspecified kidney failure), “N25” (Disorders resulting from impaired renal tubular function), “N28.0” (Other disorders of kidney and ureter, not elsewhere classified) , “N28.0” (Other disorders of kidney and ureter, not elsewhere classified) , “N28.0” (Ischaemia and infarction of kidney) , “N28.1” (Cyst of kidney), local codes for kidney diseases (20066251, 20086026, 20066317, 20066250, 20066247, 20054950, 20066253, 20066324 and 20072079) under “N28.8” (Other specified disorders of kidney and ureter) and local codes for kidney diseases (20090165, 20066327, 2066223, 20066255, 20098883, 20092361, 20054379 and 20098688) under “N28.9” (Disorder of kidney and ureter, unspecified).

Note: we used the codes which are the same as those used for “Abnormal kidney function” of HAS-BLED as the original description (J Am Coll Cardiol 2011; 19: 395-401) for “severe renal disease” (eGFR < 30 mL/min or dialysis-dependent) was judged to be roughly equivalent to a group of disorders under those ICD-codes.

Age (≥75) (2): estimated from age at the first diagnosis of NVAF (not positive in the current study as those aged 75 years old or older are not included in the study population).

Bleeding (1): All local diagnosis codes under ICD-10 code “D62” (Acute posthaemorrhagic anaemia), “I61” (Intracerebral haemorrhage), “I62” (Other nontraumatic intracranial haemorrhage), “I85.0” (Oesophageal varices with bleeding), “J94.2” (Haemothorax), “K22.6” (Gastro-oesophageal laceration-haemorrhage syndrome), “K25.0”, “K25.2”, “K25.4”, “K25.6” (Gastric ulcer with bleeding), “K26.0”, “K26.2”, “K26.4”, “K26.6” (Duodenal ulcer with bleeding), “K27.0”, “K27.2”, “K27.4”, “K75.6” (Peptic ulcer, site unspecified with bleeding), “K28.0”, “K28.2”, “K28.4”, “K28.6” (Gastrojejunal ulcer with bleeding), “K29.0” (Acute haemorrhagic gastritis) , “K62.5” (Haemorrhage of anus and rectum), “K66.1” (Haemoperitoneum), “K92.0” (Haematemesis), “K92.1” (Melaena), “K92.2” (Gastrointestinal haemorrhage, unspecified),　“M25.0” (Haemarthrosis), “N42.1” (Congestion and haemorrhage of prostate), “N92.0” (Excessive and frequent menstruation with regular cycle), “N92.1” (Excessive and frequent menstruation with irregular cycle), “R04” (Haemorrhage from respiratory passages), “R31” (Unspecified haematuria), “R58” (Haemorrhage, not elsewhere classified), “S06.4” (Epidural haemorrhage), “S06.5” (Traumatic subdural haemorrhage), “S06.6” (Traumatic subarachnoid haemorrhage) and “S06.8” (Other intracranial injuries).

Hypertension (1): All local diagnosis codes under ICD-10 code “I10” (Essential hypertension), “I11” (Hypertensive heart disease), “I12” (Hypertensive renal disease), “I13” (Hypertensive heart and renal disease) and “I15” (Secondary hypertension).

Charlson Comorbidity Index

Local diagnosis codes under ICD-10 codes used to estimate Charlson Comorbidity Index specified in Sundararajan V et al. (J Clin Epidemiol 2004; 57: 1288-94).
