# Supplementary material for: The use of anticoagulants in patients with non-valvular atrial fibrillation between 2005 and 2014: A drug utilization study using claims data in Japan
Source: PLoS One. 2018 Sep 5;13(9):e0203380. doi: 10.1371/journal.pone.0203380 (PMC6124773; doi:10.1371/journal.pone.0203380)
Supplement: S2 File — Table A. Young (20–64 years old) patients (N = 7,451). Table B. Old (65–74 years old) patients (N = 1,883). (DOCX) [file pone.0203380.s002.docx]

**S2 File.**

**Table A Young patients (N=7,451)**

| Variables | | Incident NVAF ^a^ | | Prevalent NVAF ^b^ | | | | |
| --- | --- | --- | --- | --- | --- | --- | --- | --- |
| Population,　N | | 2,942 | | 4,509 | | | | |
| Age, years (SD) | | 49.8 (10.1) | | 53.6 (9.3) | | | | |
| Male, (%) | | 66.7% | | 77.4% | | | | |
| Follow-up period, years, Mean (SD) | | 5.3 (2.5) | | 2.9 (2.0) | | | | |
| Median (IQR) | | 4.6 (3.5-8.2) | | 2.3 (1.3-4.2) | | | | |
| Missing, N(%) ^c^ | | 525 (17.8%) | | 1,614 (35.8%) | | | | |
| Comorbidities (ICD-10 code), N (%) ^d^ | | | | | | | | |
| Malignant neoplasms (C0-C9) | 158 (5.4%) | | | | 266 (5.9%) | | | |
| Thyrotoxicosis (E05) | 59 (2.0%) | | | | 357 (7.9%) | | | |
| Diabetes mellitus (E10-E14) | 496 (16.9%) | | | | 1,400 (31.0%) | | | |
| Dyslipidemia (E78) | 694 (23.6%) | | | | 1,910 (42.4%) | | | |
| Hyperuricemia (E790)/ Gout (M10) | 296 (10.1%) | | | | 962 (21.3%) | | | |
| Depression (F32) | 114 (3.9%) | | | | 223 (4.9%) | | | |
| Insomnia (G47.0) | 270 (9.2%) | | | | 701 (15.5%) | | | |
| Hypertension (I10) | 906 (30.8%) | | | | 2,765 (61.3%) | | | |
| Ischemic heart disease (I20, I21) | 353 (12.0%) | | | | 1,322 (29.3%) | | | |
| Heart failure (I50) | 270 (9.2%) | | | | 1,819 (40.3%) | | | |
| Cerebral hemorrhage (I60, I61) | 16 (0.5%) | | | | 33 (0.7%) | | | |
| Cerebral infarction (I63)/ TIA (G45.9) | 102 (3.5%) | | | | 511 (11.3%) | | | |
| Acute upper respiratory infection (J06.9) | 448 (15.2%) | | | | 733 (16.3%) | | | |
| Allergic Rhinitis (J30.4) | 607 (20.6%) | | | | 993 (22.0%) | | | |
| Asthma (J45) | 289 (9.8%) | | | | 428 (9.5%) | | | |
| Gastrointestinal Bleeding  (K22.6, K25.0, K25.4, K29.0) | 29 (1.0%) | | | | 56 (1.2%) | | | |
| Unspecified liver disease (K76.9) | 173 (5.9%) | | | | 391 (8.7%) | | | |
| Unspecified dermatitis (L30.9) | 381 (13.0%) | | | | 617 (13.7%) | | | |
| Chronic Kidney Disease (N18) | 51 (1.7%) | | | | 122 (2.7%) | | | |
| Co-medication, N (%) ^d^ | | | | | | | | |
| Antineoplastic drug | | | 44 (1.5%) | | | 67 (1.5%) | | |
| Antihyperthyroid drug | | | 30 (1.0%) | | | 195 (4.3%) | | |
| Oral antidiabetic drug | | | 174 (5.9%) | | | 510 (11.3%) | | |
| Insulin | | | 71 (2.4%) | | | 180 (4.0%) | | |
| Antihyperuricemic drug | | | 216 (7.3%) | | | 719 (15.9%) | | |
| Lipid lowering drug | | | 396 (13.5%) | | | 1,151 (25.5%) | | |
| Antidepressant | | | 86 (2.9%) | | | 170 (3.8%) | | |
| Anxiolytic drug | | | 267 (9.1%) | | | 642 (14.2%) | | |
| Antihypertensive drug | | | 945 (32.1%) | | | 3,162 (70.1%) | | |
| Loop diuretic | | | 126 (6.0%) | | | 587 (14.2%) | | |
| Antihistamine | | | 772 (26.2%) | | | 1,186 (26.3%) | | |
| Inhaled steroid | | | 176 (6.0%) | | | 269 (6.0%) | | |
| Anti-peptic ulcer drug | | | 1,221 (41.5%) | | | 2,300 (51.0%) | | |
| NSAID | | | 1,166 (39.6%) | | | 1,798 (39.9%) | | |
| Treatment for NVAF ^e^ | | | | | | | | |
| Warfarin | | | 627 (21.3%) | | | | | 1,756 (38.9%) |
| DOAC | | | 405 (13.8%) | | | | | 232 (5.1%) |
| Antiplatelet | | | 592 (20.1 %) | | | | | 1,285 (28.5%) |
| Catheter ablation | | | 166 (5.6%) | | | | | 120 (2.7%) |
| Maze procedure | | | 15 (0.5%) | | | | | 6 (0.1%) |
| Drugs for rhythm control (iv) ^f^ | | | 332 (11.3%) | | | | | 157 (3.5%) |
| Drugs for rhythm control (oral) ^g^ | | | 1,039 (35.3%) | | | | | 1,360 (30.2%) |
| Scores for the risk of stroke, bleeding and burden comorbidities | | | | | | | | |
| CHADS_2_ score (≥2) | | | 771 (26.2%) | | | | 2,717 (60.3%) | |
| CHA_2_DS_2_VASc score (≥2) | | | 929 (31.6%) | | | | 3,045 (67.5%) | |
| HAS-BLED score (≥3) | | | 290 (9.9%) | | | | 830 (18.4%) | |
| ATRIA score (≥3) | | | 299 (10.2%) | | | | 703 (15.6%) | |
| CCI score (≥3) | | | 902 (30.7%) | | | | 2,097 (46.5%) | |

Abbreviations: NVAF, non-valvular atrial fibrillation; SD, standard deviation; IQR, interquartile range; ICD-10, 10th revision of the International Statistical Classification of Diseases; TIA, transient ischemic attack; NSAID, non-steroidal anti-inflammatory drug; DOAC, direct oral anticoagulant; iv, intravenous.

a. Patients who had the first diagnosis code of NVAF 1 year or more after the first day of observation period.

b. Patients who had the first diagnosis code of NVAF within 1 year after the first day of observation period.

c. Patients whose last day of observation was earlier than the last day when the data acquisition from the insurance ended.

d. Diagnoses or drugs recorded during 1 year preceding the first diagnosis code of NVAF (for incident NVAF patients) or during the first 1 year (for prevalent NVAF patients).

e. Treatment for NVAF recorded in the first 6 months after the first diagnosis code of NVAF where one patient may be counted twice or more for different treatments.

f. Solution of aprindine, amiodarone, cibenzoline, pilsicainide, flecainide or disopyramide for intravenous use.

g Tablet or capsule of aprindine, amiodarone, cibenzoline, pilsicainide, flecainide, propafenone, bepridil or disopyramide for oral use.

**Table B Old patients (N=1,883*)**

| Variables | | | Incident NVAF ^a^ | | Prevalent NVAF ^b^ | | | |
| --- | --- | --- | --- | --- | --- | --- | --- | --- |
| Population,　N | | | 410 | | 994 | | | |
| Age, years (SD) | | | 69.2 (2.8) | | 69.1 (2.9) | | | |
| Male, (%) | | | 58.3% | | 60.5% | | | |
| Follow-up period, years, Mean (SD) | | | 4.2 (1.9) | | 2.4 (1.6) | | | |
| Median (IQR) | | | 4.0 (2.9-5.3) | | 2.2 (1.2-3.2) | | | |
| Missing, N(%) ^c^ | | | 155 (37.8%) | | 505 (50.8%) | | | |
| Comorbidities (ICD-10 code), N (%) ^d^ | | |  | | | | | |
| Malignant neoplasms (C0-C9) | | | 51 (12.4%) | | | | 129 (13.0%) | |
| Thyrotoxicosis (E05) | | | 6 (1.5%) | | | | 52 (5.2%) | |
| Diabetes mellitus (E10-E14) | | | 125 (30.5%) | | | | 416 (41.9%) | |
| Dyslipidemia (E78) | | | 190 (46.3%) | | | | 504 (50.7%) | |
| Hyperuricemia (E790)/ Gout (M10) | | | 61 (14.9%) | | | | 218 (21.9%) | |
| Depression (F32) | | | 21 (5.1%) | | | | 57 (5.7%) | |
| Insomnia (G47.0) | | | 94 (22.9%) | | | | 267 (26.9%) | |
| Hypertension (I10) | | | 250 (61.0%) | | | | 766 (77.1%) | |
| Ischemic heart disease (I20, I21) | | | 93 (22.7%) | | | | 360 (36.2%) | |
| Heart failure (I50) | | | 68 (16.6%) | | | | 455 (45.8%) | |
| Cerebral hemorrhage (I60, I61) | | | 5 (1.2%) | | | | 9 (0.9%) | |
| Cerebral infarction (I63)/ TIA (G45.9) | | | 38 (9.3%) | | | | 201 (20.2%) | |
| Acute upper respiratory infection (J06.9) | | | 61 (14.9%) | | | | 176 (17.7%) | |
| Allergic Rhinitis (J30.4) | | | 104 (25.4%) | | | | 218 (21.9%) | |
| Asthma (J45) | | | 42 (10.2%) | | | | 117 (11.8%) | |
| Gastrointestinal Bleeding  (K22.6, K25.0, K25.4, K29.0) | | | 2 (0.5%) | | | | 28 (2.8%) | |
| Unspecified liver disease (K76.9) | | | 31 (7.6%) | | | | 106 (10.7%) | |
| Unspecified dermatitis (L30.9) | | | 88 (21.5%) | | | | 188 (18.9%) | |
| Chronic Kidney Disease (N18) | | | 12 (2.9%) | | | | 26 (2.6%) | |
| Co-medication, N (%) ^d^ | | | | | | | | |
| Antineoplastic drug | | | | 7 (1.7%) | | | | 20 (2.0%) |
| Antihyperthyroid drug | | | | 2 (0.5%) | | | | 24 (2.4%) |
| Oral antidiabetic drug | | | | 55 (13.4%) | | | | 157 (15.8%) |
| Insulin | | 19 (4.6%) | | | | 57 (5.7%) | | |
| Antihyperuricemic drug | | 43 (10.5%) | | | | 160 (16.1%) | | |
| Lipid lowering drug | | 115 (28.0%) | | | | 342 (34.4%) | | |
| Antidepressant | | 8 (2.0%) | | | | 35 (3.5%) | | |
| Anxiolytic drug | | 78 (19.0%) | | | | 220 (22.1%) | | |
| Antihypertensive drug | | 248 (60.5%) | | | | 776 (78.1%) | | |
| Loop diuretic | | 33 (8.0%) | | | | 205 (20.6%) | | |
| Antihistamine | | 132 (32.2%) | | | | 258 (26.0%) | | |
| Inhaled steroid | | 33 (8.0%) | | | | 42 (4.2%) | | |
| Anti-peptic ulcer drug | | 219 (53.4%) | | | | 601 (60.5%) | | |
| NSAID | | 180 (43.9%) | | | | 397 (39.9%) | | |
| Treatment for NVAF ^e^ | | | | | | | | |
| Warfarin | 133 (32.4%) | | | | | 486 (48.9%) | | |
| DOAC | 91 (22.2%) | | | | | 51 (5.1%) | | |
| Antiplatelet | 133 (32.4%) | | | | | 361 (36.3%) | | |
| Catheter ablation | 11 (2.7%) | | | | | 6 (0.6%) | | |
| Maze procedure | 1 (0.2%) | | | | | 0 (0%) | | |
| Drugs for rhythm control (iv) ^f^ | 39 (9.5%) | | | | | 23 (2.3%) | | |
| Drugs for rhythm control (oral) ^g^ | 123 (30.0%) | | | | | 235 (23.6%) | | |
| Scores for the risk of stroke, bleeding and burden comorbidities | | | | | | | | |
| CHADS_2_ score (≥2) | 220 (53.7%) | | | | | | 765 (77.0%) | |
| CHA_2_DS_2_VASc score (≥2) | 357 (87.1%) | | | | | | 963 (96.9%) | |
| HAS-BLED score (≥3) | 219 (53.4%) | | | | | | 685 (68.9%) | |
| ATRIA score (≥3) | 71 (17.3%) | | | | | | 197 (19.8%) | |
| CCI score (≥3) | 223 (54.4%) | | | | | | 654 (65.8%) | |

Abbreviations: NVAF, non-valvular atrial fibrillation; SD, standard deviation; IQR, interquartile range; ICD-10, 10th revision of the International Statistical Classification of Diseases; TIA, transient ischemic attack; NSAID, non-steroidal anti-inflammatory drug; DOAC, direct oral anticoagulant; iv, intravenous.

a. Patients who had the first diagnosis code of NVAF 1 year or more after the first day of observation period.

b. Patients who had the first diagnosis code of NVAF within 1 year after the first day of observation period.

c. Patients whose last day of observation was earlier than the last day when the data acquisition from the insurance ended.

d. Diagnoses or drugs recorded during 1 year preceding the first diagnosis code of NVAF (for incident NVAF patients) or during the first 1 year (for prevalent NVAF patients).

e. Treatment for NVAF recorded in the first 6 months after the first diagnosis code of NVAF where one patient may be counted twice or more for different treatments.

f. Solution of aprindine, amiodarone, cibenzoline, pilsicainide, flecainide or disopyramide for intravenous use.

g Tablet or capsule of aprindine, amiodarone, cibenzoline, pilsicainide, flecainide, propafenone, bepridil or disopyramide for oral use.

*1,883 patients include of 1,404 who were 65-74 years old when they had the first diagnosis code of NVAF and 479 who became 65 years old during the observation period.
