# Supplementary material for: The use of anticoagulants in patients with non-valvular atrial fibrillation between 2005 and 2014: A drug utilization study using claims data in Japan
Source: PLoS One. 2018 Sep 5;13(9):e0203380. doi: 10.1371/journal.pone.0203380 (PMC6124773; doi:10.1371/journal.pone.0203380)
Supplement: S3 File — Figure A. Young (20–64 years old) patients (N = 7,451). Figure B. Old (65–74 years old) patients (N = 1,883). (DOCX) [file pone.0203380.s003.docx]

**S3 File.**

**Fig A Young patients (N=7,451)**

**(b)**

**Proportion of Prevalence of NVAF (%)**

**Fig B Old patients (N=1,883*)**

**(b)**

**Proportion of Prevalence of NVAF (%)**

*1,883 patients include of 1,404 who were 65-74 years old when they had the first diagnosis code of NVAF and 479 who became 65 years old during the observation period.
