# Supplementary material for: The use of anticoagulants in patients with non-valvular atrial fibrillation between 2005 and 2014: A drug utilization study using claims data in Japan
Source: PLoS One. 2018 Sep 5;13(9):e0203380. doi: 10.1371/journal.pone.0203380 (PMC6124773; doi:10.1371/journal.pone.0203380)
Supplement: S4 File — Table A. Young (20–64 years old) patients (N = 7,451). Table B. Old (65–74 years old) patients (N = 1,883). (DOCX) [file pone.0203380.s004.docx]

**S4 File.**

**Table A Young patients (N=7,451)**

**The results of the segmented regression analysis**

| Variables | Estimate | p value |
| --- | --- | --- |
| Intercept (β_0_) | 37.5 | <0.0001 |
| Baseline trend (β_1_) | 0.435 | <0.0001 |
| Level change at DOAC advent (β_2_) | - 0.083 | 0.94 |
| Trend change after DOAC advent (β_3_) | 0.149 | 0.44 |

The data for the proportion of the prevalence of patients with NVAF and anticoagulant in the young patients shown in S2 File was analyzed.

**Table B Old patients (N=1,883*)**

**The results of the segmented regression analysis**

| Variables | Estimate | p value |
| --- | --- | --- |
| Intercept (β_0_) | 39.3 | <0.0001 |
| Baseline trend (β_1_) | 0.726 | <0.0001 |
| Level change at DOAC advent (β_2_) | 0.04 | 0.98 |
| Trend change after DOAC advent (β_3_) | 0.12 | 0.62 |

The data for the proportion of the prevalence of patients with NVAF and anticoagulant in the old patients shown in S2 File was analyzed.

*1,883 patients consist of 1,404 who were 65-74 years old when they had the first diagnosis code of NVAF and 479 who became 65 years old during the observation period.
