# Supplementary material for: The use of anticoagulants in patients with non-valvular atrial fibrillation between 2005 and 2014: A drug utilization study using claims data in Japan
Source: PLoS One. 2018 Sep 5;13(9):e0203380. doi: 10.1371/journal.pone.0203380 (PMC6124773; doi:10.1371/journal.pone.0203380)
Supplement: S5 File — Figure A. Young (20–64 years old) patients (N = 2,403). Figure B. Old (65–74 years old) patients (N = 286). Figure A_a and Figure B_b show the incidence (/100,000 person-years) standardized to Japanese population as of the 2012 census. Figure A_b and Figure B_b show the proportion of the incidence subdivided by the drug started within 6 months after the first diagnosis of NVAF (%). (DOCX) [file pone.0203380.s005.docx]

**S5 File.**

**Fig A Young patients (N=2,403)**

**Proportion of Patients**

**with Incident NVAF (%)**

**(b)**

**Fig B Old patients (N=286)**

**(b)**

**Proportion of Patients**

**with Incident NVAF (%)**
