# Supplementary material for: The use of anticoagulants in patients with non-valvular atrial fibrillation between 2005 and 2014: A drug utilization study using claims data in Japan
Source: PLoS One. 2018 Sep 5;13(9):e0203380. doi: 10.1371/journal.pone.0203380 (PMC6124773; doi:10.1371/journal.pone.0203380)
Supplement: S6 File — Table A. Young (20–64 years old) patients (N = 7,451). Table B. Old (65–74 years old) patients (N = 1,883). (DOCX) [file pone.0203380.s006.docx]

**S6 File.**

**Table A Young patients (N=7,451)**

| Class/Drug | N | Duration ^a^  (years) | MPR ^b^  (%) |
| --- | --- | --- | --- |
| Incident NVAF |  | | |
| antiplatelet | 181 | 3.1 | 35.7 |
| warfarin | 513 | 2.7 | 55.9 |
| DOAC | 270 | 1.2 | 58.3 |
| Prevalent NVAF |  | | |
| antiplatelet | 485 | 3.3 | 63.2 |
| warfarin | 1,065 | 3.0 | 77.9 |
| DOAC | 120 | 1.4 | 69.0 |

DOAC, direct oral anticoagulant

a. Time duration from the first dispensing of the drug to the last day of observation (denominator).

b. Total days-supply (numerator) divided by the total of the duration from the first dispensing to the last day of observation (denominator).

**Table B Old patients (N=1,883*)**

| Class/Drug | N | Duration ^a^  (years) | MPR ^b^  (%) |
| --- | --- | --- | --- |
| Incident NVAF |  | | |
| antiplatelet | 34 | 2.5 | 55.6 |
| warfarin | 95 | 2.0 | 63.8 |
| DOAC | 47 | 1.1 | 62.6 |
| Prevalent NVAF |  | | |
| antiplatelet | 114 | 2.6 | 60.2 |
| warfarin | 282 | 2.7 | 80.1 |
| DOAC | 18 | 1.5 | 79.2 |

DOAC, direct oral anticoagulant

a. Time duration from the first dispensing of the drug to the last day of observation (denominator).

b. Total days-supply (numerator) divided by the total of the duration from the first dispensing to the last day of observation (denominator).

*1,883 patients consist of 1,404 who were 65-74 years old when they had the first diagnosis code of NVAF and 479 who became 65 years old during the observation period.
