# Supplementary material for: The use of anticoagulants in patients with non-valvular atrial fibrillation between 2005 and 2014: A drug utilization study using claims data in Japan
Source: PLoS One. 2018 Sep 5;13(9):e0203380. doi: 10.1371/journal.pone.0203380 (PMC6124773; doi:10.1371/journal.pone.0203380)
Supplement: S7 File — Table A. Young (20–64 years old) patients (N = 2,403). Table B. Old (65–74 years old) patients (N = 286). (DOCX) [file pone.0203380.s007.docx]

**S7 File.**

**Table A Young patients (N=2,403)**

| Period | January 2006 – June 2008 | | | July 2008 – December 2010 | | | January 2011 – September 2013 | | | |
| --- | --- | --- | --- | --- | --- | --- | --- | --- | --- | --- |
| Population, N | 461,938 | | | 1,157,143 | | | 1,957,150 | | | |
| Age, years (SD) | 38.5(11.5) | | | 38.5 (11.6) | | | 39.2 (11.7) | | | |
| Male, % | 58.7% | | | 50.6% | | | 52.0% | | | |
| New users | No AC/AP | AP | Warfarin | No AC/AP | AP | Warfarin | No AC/AP | AP | Warfarin | DOAC |
| Number | 204 | 54 | 85 | 356 | 58 | 165 | 879 | 69 | 263 | 270 |
| Age, years (SD) | 48.0(9.5) | 51.7(8.6) | 51.7(7.9) | 46.1(11.1) | 50.6(9.9) | 53.0(9.3) | 48.5(10.3) | 52.0(9.6) | 52.1(8.5) | 53.9(8.1) |
| Male, % | 79.9% | 90.7% | 89.4% | 76.7% | 86.2% | 86.1% | 76.6% | 87.0% | 85.6% | 84.4% |
| CHADS_2_ score (≥2), N  % | 48  23.5% | 20  37.0% | 34  40.0% | 107  30.1% | 27  46.6% | 79  47.9% | 203  23.1% | 28  40.6% | 129  49.0% | 87  32.2% |
| CHA_2_DS_2_VASc score (≥2), N  % | 69  33.8% | 23  42.6% | 39  45.9% | 147  41.3% | 32  55.2% | 92  55.8% | 273  31.1% | 30  43.5% | 146  55.5% | 102  37.8% |
| HAS-BLED score (≥3), N  % | 11  5.4% | 8  14.8% | 8  9.4% | 28  7.9% | 9  15.5% | 20  12.1% | 53  6.0% | 8  11.6% | 33  12.5% | 10  3.7% |
| ATRIA score (≥3), N  % | 16  7.8% | 4  7.4.% | 6  7.1% | 32  9.0% | 6  10.3% | 17  10.3% | 72  8.2% | 7  10.1% | 29  11.0% | 19  7.0% |
| CCI score (≥3), N  % | 61  29.9% | 16  29.6.% | 27  31.8% | 114  32.0% | 23  39.7% | 65  39.4% | 223  25.4% | 27  39.1% | 82  31.2% | 63  23.3% |

NVAF: non-valvular atrial fibrillation, SD: standard deviation, No AC/AP: no anticoagulant/antiplatelet; AP: antiplatelet; DOAC: direct oral anticoagulant; CCI: Charlson Comorbidity Index.

**Table B Old patients (N=286)**

| Period | January 2006 – June 2008 | | | July 2008 – December 2010 | | | January 2011 – September 2013 | | | |
| --- | --- | --- | --- | --- | --- | --- | --- | --- | --- | --- |
| Population, N | 34,107 | | | 68,450 | | | 102,705 | | | |
| Age, years (SD) | 68.9(2.4) | | | 68.7 (2.4) | | | 68.7 (2.4) | | | |
| Male, % | 52.9% | | | 52.1% | | | 52.3% | | | |
| New users | No AC/AP | AP | Warfarin | No C/AP | AP | Warfarin | No AC/AP | AP | Warfarin | DOAC |
| Number | 15 | 9 | 10 | 27 | 11 | 35 | 68 | 14 | 50 | 47 |
| Age, years (SD) | 69.9(2.6) | 69.9(2.7) | 70.6(2.5) | 69.4(3.1) | 69.5(2.3) | 69.4(3.0) | 69.2(2.8) | 69.6(2.5) | 69.4(2.8) | 69.5(2.8) |
| Male, % | 40.0% | 22.2% | 50.0% | 33.3% | 18.2% | 65.7% | 63.2% | 42.9% | 68.0% | 70.2% |
| CHADS_2_ score (≥2), N  % | 2  13.3% | 5  55.6% | 5  50.0% | 11  40.7% | 6  54.5% | 14  40.0% | 19  27.9% | 6  42.9% | 23  46.0% | 17  36.2% |
| CHA_2_DS_2_VASc score (≥2), N  % | 12  80.0% | 9  100% | 8  80.0% | 23  85.2% | 10  90.9% | 27  77.1% | 45  66.2% | 13  92.9% | 35  70.0% | 33  70.2% |
| HAS-BLED score (≥3), N  % | 1  6.7% | 4  44.4% | 2  20.0% | 8  29.6% | 6  54.5% | 9  25.7% | 20  29.4% | 5  35.7% | 19  38.0% | 11  23.4% |
| ATRIA score (≥3), N  % | 2  13.3% | 1  11.1.% | 0  0% | 2  7.4% | 2  18.2% | 4  11.4% | 9  13.2% | 2  14.3% | 7  14.0% | 5  10.6% |
| CCI score (≥3), N  % | 2  13.3% | 6  66.7% | 3  30.0% | 9  33.3% | 5  45.5% | 16  45.7% | 28  41.2% | 5  35.7% | 23  46.0% | 19  40.4% |

NVAF: non-valvular atrial fibrillation, SD: standard deviation, No AC/AP: no anticoagulant/antiplatelet; AP: antiplatelet; DOAC: direct oral anticoagulant; CCI: Charlson Comorbidity Index.
