# Supplementary material for: The use of anticoagulants in patients with non-valvular atrial fibrillation between 2005 and 2014: A drug utilization study using claims data in Japan
Source: PLoS One. 2018 Sep 5;13(9):e0203380. doi: 10.1371/journal.pone.0203380 (PMC6124773; doi:10.1371/journal.pone.0203380)
Supplement: S8 File — Table A. Young (20–64 years old) patients (N = 263). Table B. Old (65–74 years old) patients (N = 50). (DOCX) [file pone.0203380.s008.docx]

**S8 File.**

**Table A Young patients (N=263)**

|  | Patients  N | Prothrombin time  measurements  N | Patient  -years | Rate of  measurement  /patient-years |
| --- | --- | --- | --- | --- |
| Warfarin followed by DOAC | 39 | 397 | 28.5 | 13.9 |
| Warfarin only | 224 | 2,410 | 195.0 | 12.4 |
| Rate difference (95% CI) |  | | | 1.6 (0.1, 3.0) |

Data in 263 patients who had the first diagnosis code of NVAF in 2011 or later at least one year after the start of the observation period and then prescribed warfarin within 6 months.

**Table B Old patients (N=50)**

|  | Patients  N | Prothrombin time  measurements  N | Patient  -years | Rate of  measurement  /patient-years |
| --- | --- | --- | --- | --- |
| Warfarin followed by DOAC | 6 | 33 | 1.5 | 22.4 |
| Warfarin only | 44 | 597 | 38.1 | 15.7 |
| Rate difference (95% CI) |  | | | 6.8(-1.0, 14.6) |

Data in 50 patients who had the first diagnosis code of NVAF in 2011 or later at least one year after the start of the observation period and then prescribed warfarin within 6 months.
